# Challenges and recommendations for cancer care in the COVID-19 pandemic

**DOI:** 10.20892/j.issn.2095-3941.2020.0300

**Published:** 2020-08-15

**Authors:** Jianbo Tian, Xiaoping Miao

**Affiliations:** ^1^Department of Epidemiology and Biostatistics, Key Laboratory for Environment and Health, School of Public Health, Tongji Medical College, Huazhong University of Sciences and Technology, Wuhan 430030, China

The COVID-19 pandemic caused by severe acute respiratory syndrome coronavirus 2 (SARS-CoV-2) has spread globally. As of June 10, 2020, there were 7,127,753 confirmed cases and 407,159 deaths, and the numbers of cases were still surging across 216 countries worldwide^1^. In 2018, there were an estimated 18.1 million new cancer cases and 9.6 million cancer deaths worldwide, according to global cancer statistics^2^. Initial reports suggested that patients with cancer might be at increased risk of contracting the virus and developing COVID-19^3,4^. Indeed, patients with cancer might have immunocompromised status because of the effects of antineoplastic therapy, supportive medications such as steroids, and the immunosuppressive properties of cancer itself. Given the high transmissibility of COVID-19 and the worldwide prevalence of cancer, a systemic understanding of the clinical features and risk factors for patients with both cancer and COVID-19 is urgently needed. This knowledge should be highly valuable in identifying reliable markers of the COVID-19 disease course, and administering effective treatments for this particular patient group.

Therefore, we report the results from a cohort study conducted in 9 hospitals designated to treat patients with COVID-19 in Wuhan, China, the initial epicenter of the COVID-19 outbreak, as recently published in *The Lancet Oncology*^5^. Between January 13 and March 18, 2020, 13,077 patients with COVID-19 were admitted to the 9 hospitals in Wuhan, and 232 patients (≥ 18 years of age) with cancer and 519 patients without cancer who were statistically matched on the basis of age, sex, and comorbidities were enrolled. We found that patients with cancer were more likely to have severe or critical COVID-19 than those without cancer [148 (64%) of 232 *vs.* 166 (32%) of 519; odds ratio (OR) = 3.61 (95% CI = 2.59–5.04); *P* < 0.0001]. We noted that, at admission, patients with cancer were more likely to have symptoms of dyspnea and expectoration than patients without cancer, but were less likely to have sore throat and coryza. Moreover, CT scans revealed that ground-glass opacity and patchy shadows were more frequent in patients with cancer. When comparing the biochemical indexes of COVID-19 between patients with and without cancer, we found that pro-inflammatory cytokines, including tumor necrosis factor α (TNF-α), interleukin (IL)-6, and IL-2R, were higher in patients with cancer. The infection-related biomarkers procalcitonin and C-reactive protein were also abnormally elevated in patients with cancer. In terms of immune cells, the decline in lymphocytes, CD4^+^ T cells, CD8^+^ T cell counts, and the ratio of CD4^+^ T cells to CD8^+^ T was greater in patients with cancer. Additionally, there was greater evidence of multiple-organ damage, particularly coagulation dysfunction, in patients with than without cancer. Notably, aggravated inflammatory responses, lymphopenia, and multiple-organ damage—particularly a decreased albumin–globulin ratio (ALB/GLO) and elevated N-terminal pro-B-type natriuretic peptide (NT-proBNP)—were more pronounced in patients with cancer who had severe COVID-19 than in those with non-severe disease. Additionally, cancer patients with severe COVID-19 tended to be older, to have higher Eastern Cooperative Oncology Group (ECOG) performance status scores, and to have more advanced cancer stage than those with non-severe COVID-19. Patients with severe COVID-19 were more likely to have received chemotherapy, radiotherapy, targeted therapy, or immunotherapy than those with non-severe disease, who were more likely to have received surgical antitumor treatment. In addition, the time interval between the last chemotherapy treatment and hospital admission, and the time since cancer diagnosis also differed between patients with severe and non-severe COVID-19.

We further identified several predictors of COVID-19 severity in patients with cancer (**Figure 1**). Risk factors previously reported in patients without cancer, such as older age, elevated IL-6, procalcitonin, D-dimer, and reduced lymphocytes, were validated in patients with cancer. We also identified advanced tumor stage [OR = 2.60 (95% CI = 1.05–6.43); *P* = 0.039], higher ECOG scores [per 1-point increase; 2.80 (1.96–6.43); *P* = 0.039], elevated TNF-α [1.22 (1.01–1.47); *P* = 0.037], elevated NT-proBNP [1.65 (1.03–2.78); *P* = 0.032], lower CD4^+^ T cell counts [0.84 (0.71–0.98); *P* = 0.031], and a lower ALB/GLO ratio [0.12 (0.02–0.77); *P* = 0.024] as risk factors for COVID-19 severity in patients with cancer. Collectively, these findings suggested that patients with both cancer and COVID-19 were more likely to develop severe illness than those without cancer. The risk factors identified here may be helpful for early clinical surveillance of disease progression in patients with cancer who present with COVID-19.

Moreover, other multicenter studies have identified potential risk factors for mortality among patients with cancer and COVID-19 (**Figure 1**)^6–9^. Recently, a multicenter study in Hubei, China, which enrolled 205 patients with cancer and COVID-19, has reported that 30 (15%) patients were transferred to an intensive care unit, and 40 (20%) died in the hospital. Chemotherapy occurring within 4 weeks of symptom onset [OR = 3.51 (95% CI = 1.16–10.59); *P* = 0.026] and male sex [3.86 (1.57–9.50); *P* = 0.0033] were found to be risk factors for in-hospital death. The authors also highlighted that patients with hematological malignancies had poorer prognoses [hazard ratio for death 3.28 (95% CI = 1.56–6.91); *P* = 0.0009] than patients with solid tumors. Moreover, in data from 218 cancer patients with COVID-19 in New York, US, a total of 61 (28%) cancer patients died from COVID-19, with a case fatality rate of 37% (20/54) for hematologic malignancies and 25% (41/164) for solid malignancies^7^. The authors found that increased mortality was significantly associated with older age, multiple comorbidities, and elevated levels of D-dimer, lactate dehydrogenase and lactate in multivariable analysis. Moreover, the COVID-19 and Cancer Consortium (CCC19) summarized the data for 928 eligible patients with cancer and COVID-19 from the US, Canada, and Spain^8^ in the largest multicenter cohort study conducted to date among patients with cancer and COVID-19 worldwide. The authors reported independent factors associated with increased 30-day mortality among patients with cancer and COVID-19, including older age (per 10 years; OR = 1.84, 95% CI = 1.53–2.21), male sex (1.63, 1.07–2.48), smoking status (1.60, 1.03–2.47), number of comorbidities (two *vs.* none: 4.50, 1.33–15.28), ECOG performance status (status of 2 *vs.* 0 or 1: 3.89, 2.11–7.18), and active cancer (progression *vs.* remission: 5.20, 2.77–9.77). Interestingly, the authors found that, compared with residence in the northeastern US, residence in Canada (0.24, 0.07–0.84) or the midwestern US (0.50, 0.28–0.90) was significantly associated with decreased mortality from COVID-19. These risk factors for COVID-19 mortality were similar to those in recently published data from the UK Coronavirus Cancer Monitoring Project (UKCCMP)^9^. Of note, the 2 studies indicated that type of anticancer therapy and recent surgery were not associated with mortality due to COVID-19, in contrast to the findings in recent studies from China, possibly as a result of limited sample size^6,10^. Several other reports involving small sample sizes have focused on patients with cancer and COVID-19 and have found that cancer patients are not only more susceptible to SARS-CoV-2 infection but also are at high risk of developing more severe events than the general population^3,10,11^. To clarify the relationships among cancer, anticancer treatments, and COVID-19, larger-scale sample sizes or datasets are necessary.

All these studies together suggest that patients with cancer and COVID-19 are more likely to deteriorate and develop severe COVID-19^5^. These patients have a case fatality rate as high as 20% (**Figure 1**), a rate much higher than that in the community (1.8%–10%)^1,6,7^. Therefore, successful and effective measures for the care of patients with cancer are crucial worldwide during the COVID-19 pandemic. On the one hand, patients might be at high risk of contracting the infection, dying from COVID-19 and receiving suboptimal care, particularly in resource-constrained settings. On the other hand, if the disease is not treated appropriately, patients might be at high risk of cancer progression and death. Lee and colleagues^9^ have concluded that if effective cancer treatments are withheld from many cancer patients during the pandemic, there will be a very real risk of increasing cancer morbidity and mortality, to levels perhaps much greater than those due to COVID-19 itself. These situations raise the important issue of whether treatment plans should be initiated on schedule or delayed, and, if so, for how long? Guidance has been developed to establish priority groups and priority levels for surgery, systemic anticancer treatments, and radiotherapy. The official advice is that urgent cancer care can continue for cancers such as lung and pancreatic cancer, acute leukemia, and highly aggressive lymphoma, but other treatments should be limited and adapted, such as those for breast and thyroid cancer^12–15^. Meanwhile, for cancers not requiring acute treatment, the guidance suggests expanding telemedicine services, decreasing clinical visits, and switching to subcutaneous or oral therapies rather than intravenous ones, when possible. Moreover, He and colleagues^16^ have suggested that some anticancer drugs conventionally administered through infusion, such as etoposide and vinorelbine, may be changed to orally administered drugs if available, and that the infusion intervals of adjuvant or maintenance chemotherapy may be appropriately prolonged depending on patient condition. Second, more intensive surveillance and medical care should be considered for patients who have cancer and are admitted with COVID-19, particularly older patients or those with other comorbidities. Third, oncologists should strengthen cooperation and collegiality across borders to improve communication and work efficiency, and healthcare providers should make full preparations for a possible surge in cancer cases^16^. Fourth, robust personal protection provisions should be made for medical staff and cancer patients to avoid nosocomial cross infection.

In summary, compared with patients without cancer, patients with cancer have poorer clinical COVID-19 outcomes. Therefore, more attention should be paid to this issue, and early surveillance should be a priority. Further studies with larger sample sizes and longer-term follow-up are urgently needed to more systematically understand the effects of SARS-CoV-2 on clinical outcomes in cancer patients and to formulate comprehensive treatment measures for this special population.

## Figures and Tables

**Figure 1:**
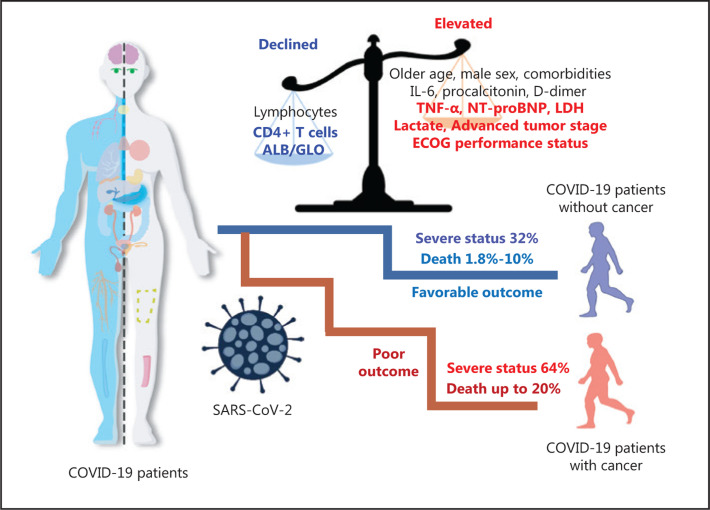
Schematic diagram of the risk factors for clinical outcomes among patients with cancer and COVID-19. Compared with those without cancer, patients with cancer had worse clinical outcomes for COVID-19. Some factors have been found to be associated with COVID-19 severity and mortality among patients with cancer. These factors include older age; male sex; comorbidities; elevated IL-6, procalcitonin, D-dimer, TNF-α, NT-proBNP, lactate dehydrogenase, and lactate; advanced tumor stage; higher ECOG performance status; and decreased lymphocytes, CD4^+^ T cells and ALB/GLO ratios.
